# Sickle Cell Disease in the Democratic Republic of Congo: Assessing Physicians’ Knowledge and Practices

**DOI:** 10.3390/tropicalmed5030127

**Published:** 2020-07-29

**Authors:** Benoît Mukinayi Mbiya, Ghislain Tumba Disashi, Béatrice Gulbis

**Affiliations:** 1 Pediatrics Department, Faculty of Medicine, University of Mbujimayi, Mbujimayi 06201, Democratic Republic of Congo; benoit.mbiya@um.ac.cd; 2 Internal Medicine Department, Faculty of Medicine, University of Mbujimayi, Mbujimayi 06201, Democratic Republic of Congo; recteur@um.ac.cd; 3 Clinical Chemistry Department-Hereditary Red Blood Cell Disorders, Laboratoire Hospitalier Universitaire de Bruxelles-Universitaire Laboratorium Brussel, Université Libre de Bruxelles, 1070 Brussels, Belgium

**Keywords:** sickle cell disease, knowledge assessment, practices, physicians, Democratic Republic of Congo

## Abstract

Background: Sickle cell disease is a major public health issue in the Democratic Republic of Congo (DRC), but it is still poorly understood by health professionals. The objective of this study was to assess the knowledge and practices of Congolese physicians treating sickle cell disease (SCD), in order to identify the areas for improvement in clinical care. Methods: This was a descriptive observational study conducted among Congolese physicians using a questionnaire. Participants were evaluated using a pre-established answer grid. Results: A total of 460 physicians participated, including 81 women (18%), with an average age of 35 years (range 25–60 years). Most physicians were general practitioners. Although self-assessment of their level of knowledge on SCD was estimated as average to good, less than half of the participants (*n* = 460; 46%) reported adequate management of vaso-occlusive crises, and only 1% of them had received specific training on SCD. Most physicians reported difficulties both in terms of diagnostic (65%) and management (79%) options of SCD patients. This study also showed that 85% of these physicians did not have access to the diagnostic tools for SCD. Conclusions: Insufficient knowledge on SCD and poor diagnostic and treatment options might contribute to increased morbidity and mortality of patients living in the DRC. Interventions aiming to improve physicians’ knowledge, patient follow-up, and treatment access are needed. Specific training alongside existing programs (HIV, malaria), early diagnosis of the disease, and the creation of patient advocacy groups should be implemented to improve SCD patient care.

## 1. Introduction

Hemoglobinopathies, mainly comprising thalassemia and sickle cell anemia, are inherited conditions. Currently, almost 5% of the world’s population has a mutation in one of the globin genes. Each year, nearly 300,000 infants worldwide are born with thalassemia syndrome (30%) or sickle cell disease (SCD) (70%) [1]. The major forms of these diseases require rapid diagnosis and treatment but are still poorly recognized by healthcare professionals, causing misdiagnosis and inadequate support, which are factors that explain the high morbidity and mortality rates in these patients [2]. It is estimated that more than 75% of sickle cell patients live in sub-Saharan Africa. The fact that the epidemiology of thalassemia in the Democratic Republic of Congo (DRC) is not known can be partly explained by the rarity of major forms [1]. In contrast, SCD is a common disease in the DRC, with 2% of newborns homozygous for hemoglobin S, representing around 40,000 births per year [3,4]. Although this figure is epidemiologically significant, the disease remains relatively unrecognized, resulting in a high mortality rate in a country with limited resources [5]. SCD is recognized as a public health problem in the DRC and benefits from national guidelines for the control and management of SCD through the national SCD program (PNLCD in French), established in 2001.

SCD is clinically expressed from the age of 3 months. Its manifestation varies in terms of its frequency and intensity, and it is classically represented as a triad of episodes of acute anemia, painful episodes of vaso-occlusive crisis (VOC), and susceptibility to infection [6].

In the DRC, high population growth, combined with significant health challenges, has created an urgent need for healthcare workers. Furthermore, human resources for healthcare are a serious problem for the sector as a whole; this is explained by a decline in professional quality, the growth of schools and universities offering cheap medical education, and few qualified staff. Many qualified physicians search for better working and living conditions and pursue their careers in South Africa. In 2012, the Ministry of Health’s Human Resources Directorate estimated that the number of DRC physicians was 5967 for a population of 70 million, which is equivalent to 1 physician per 11,731 inhabitants [7]. We did not find reliable and up-to-date data on the total number of physicians and their distribution, according to age or sex or among provinces. This is explained by the recent mass exodus of physicians to Southern Africa, by the number of physicians working in the private sector, and by the migration of health personnel within the country [8,9]. There are three major traditional universities for training physicians (University of Kinshasa, Lubumbashi University, and the University of Kisangani), and the emergence of private universities or under-agreement medical training is not sufficient to meet healthcare needs. This is partly explained by the use of training programs that are not adapted to local needs, a poorly organized training framework, a lack of appropriate internship places, a qualitative and quantitative shortage of teachers, and a lack of model schools for testing and adapting the training programs [8].

To overcome some of the shortcomings of physicians’ knowledge on pathologies such as HIV/AIDS or malaria, national HIV and malaria programs receiving support from the World Health Organization (WHO) regularly organize capacity building training for caregivers and physicians, which results in the improved care and management of these diseases. Significant efforts are being made to integrate vertical disease control programs into the primary healthcare package, particularly in the fight against HIV and malaria [10,11].

Most Congolese have no access to quality healthcare. Moreover, insufficient knowledge among physicians might contribute to the high mortality and morbidity rates related to sickle cell disease. This assumption led us to evaluate the knowledge and practices of Congolese physicians concerning SCD patient care and to propose concrete actions to improve it.

## 2. Methods

This was a descriptive observational study conducted on a population that included physicians, who predominantly practiced in five cities within the DRC. To collect the information, a questionnaire was delivered online or directly by hand, via existing health structures or existing networks in Kinshasa (DRC capital), Lubumbashi (Haut Katanga), Mbuji-Mayi (Kasai-Oriental), Bukavu (South Kivu), Kananga (Kasai-central), and other cities. The selected cities are the most heavily populated in the DRC [12] and contain, at minimum, a university medical school.

The study comprised any general practitioner (GP) or specialist practicing in the DRC and registered in the National Council of the Order of Physicians who agreed to participate in the study. Any doctor registered in the National Council of the Order of Physicians obtains a serial number (CNOM in French) that authorizes him or her to practice medicine in the DRC.

Questionnaires with less than 50% of the questions answered were excluded from the analysis.

The questionnaire was developed in collaboration with the medical faculty of the University of Mbujimayi in the DRC and the Department of Clinical Chemistry at the Université Libre de Bruxelles (ULB). It was trialed on 30 physicians in Mbujimayi. Answers from the trial were used to improve the questionnaire, especially for the use of unequivocal formulations. The finalized questionnaire was submitted to the Department of Epidemiology and Biostatistics (School of Public Health, ULB) for validation.

The questionnaire comprised 57 questions divided into five groups—(1) physician profile; (2) SCD clinical practice; (3) diagnosis and clinical management of hemoglobinopathies; (4) organizational management of SCD, and (5) concerns about hemoglobinopathies and self-assessment of knowledge of these conditions.

Questions were either closed (dichotomous or nominal) or open. Open questions were evaluated as either a right answer (the answer was correct according to a grid of pre-established responses) or an inappropriate response (the answer was irrelevant, inadequate, or wrong), and self-assessment of knowledge on hemoglobinopathies was recorded through a scale of Likert values [13]. The self-assessment scale comprised five numeric values ranging from 1 to 5. The scale was 1 = poor knowledge, 2 = insufficient knowledge, 3 = average knowledge, 4 = good knowledge, and 5 = very good knowledge.

Recommendations for the management of vaso-occlusive crises were defined by the prescription of analgesics (paracetamol, non-steroidal anti-inflammatory, or morphine), hyperhydration, and the treatment of the factor favoring the crisis (infections—malaria, sepsis, etc.), while recommendations for the management of acute anemia were defined by the prescription of red blood cell transfusions.

A large city was defined by its urban landscape and the presence of an international airport directly connected to foreign countries, while a remote city was defined by a semi-rural landscape, located inside the country without direct contact with foreign countries.

No instructions were given that could guide responses. In the case of a non-response, doctors were contacted repeatedly by either mail, phone, SMS, Facebook, LinkedIn, or direct contact.

## 3. Statistical Analysis

Statistical analysis of the collected data was performed using the EPI Info software (version 5.0, CDC, Atlanta, GA, USA, 2008). The data, as appropriate, are represented by the mean and confidence interval at 95% for numerical parameters and absolute or relative frequency for temperamental parameters. The chi-square test was used to compare the level of knowledge of the participants, with a *p*-value < 0.05 considered to be significant.

## 4. Results

### 4.1. Physician Profile and Practice

Questionnaires were sent out from June 2017 to September 2018 to 956 physicians, and 475 responses were received. Of these, 15 were excluded—7 were from physicians practicing outside the DRC, 3 did not include the serial number of the doctor, and 5 had responses to less than 50% of the questions.

The response rate was 48% (460/956). The median age was 35 years (range 25–60 years), and the minority of participants (18%; 81/460) were women.

The geographical distribution of participants by percentage was 35% in Mbujimayi, 13% in Kinshasa, 12% in Lubumbashi, 22% in Kananga, 12% in Bukavu, and 6% in other towns. Most participants were general practitioners (82.4%), and a few were specialists, i.e., pediatricians (4.1%), public health specialists (4.1%), or other specialists (9.4%). Most of them (86%) had a clinical practice, while a few of them (1%) had taken a diploma course on major SCD syndromes. Most of them (83%) had less than 10 years of clinical experience, and a few (17%) had more than 10 years experience. The majority (52.2%) worked in a General Reference Hospital (HGR), and the others (26% and 13%) worked in health centers or a private hospital, respectively. Sickle cell disease clinical practice and knowledge among the physicians are described in Table 1.

### 4.2. Diagnosis and Clinical Management of SCD

Physicians reported that the circumstances of SCD diagnosis were mainly clinical (80%). The Emmel test (sickling test) [14,15] was the most frequently requested test (83%). Diagnostic testing was requested most often at the time of disease complications (99%) and rarely for neonatal screening (1%). Approximately two-thirds of the physicians (65%) reported difficulty performing hemoglobin electrophoresis due to a lack of equipment. The vast majority of the participants (91%) reported the sole disease in this group to be sickle cell anemia (homozygosity for hemoglobin S), and 99% were not aware of the other syndromes (thalassemia, HbSC, etc.). The different SCD syndromes encountered are presented in Table 1.

Less than half (44%) followed recommendations for the management of VOC and prescribed analgesics and hydration while treating the condition triggering the crisis, typically infections such as malaria. Episodes of acute anemia were treated with red blood cell transfusions by 86% of physicians. After managing a sickle cell crisis, 24% of physicians referred the patient to an appropriate support center for future monitoring.

Few physicians (9%) prescribed hydroxyurea, and only 26% of them thought that hydroxyurea was available in a local pharmacy. Most of them were not aware of the price of hydroxyurea (74%), and they responded that it is not affordable for their patients (91%). The majority (95%) also reported that the therapeutic means for SCD management are not sufficient, and most of them (79%) suggested that conditions should be implemented to improve SCD patient care.

The physicians interviewed noted certain concerns—many were in terms of training, such as the lack of local training for a university degree in sickle cell syndrome (79%), but concerns were also related to patient follow-up, the difficulty of organizing regular medical follow-up, and the rigorous application of management recommendations.

### 4.3. Self-Assessment and Concerns

For most physicians (84%), hemoglobinopathies are a concern in their clinical practice, and they (79%) reported that there are conditions that need to be met to improve services and the treatment burden of sickle cell patients. Training on major sickle cell syndromes, the availability and accessibility of hydroxyurea, and diagnostic means (hemoglobin electrophoresis) were the aspects most often reported to need improvement.

Self-assessment of the level of SCD knowledge was reported as between average and good (Table 2).

Compared with those practicing in remote cities of the DRC (138/348; 40%) or having a clinical practice of less than 10 years (162/382; 42%), physicians practicing in large cities or having a practice of more than 10 years felt that they had better knowledge of the management of vaso-occlusive crises, with values of 66% (74/112) and 65% (51/78) (*p* = 0.000), respectively.

## 5. Discussion

Sickle cell disease is a chronic disease with multi-organ involvement. Its management is complex, particularly in the DRC, where access to care, the low socioeconomic level, and other factors such as malaria play a significant role in its morbidity and mortality rates. For optimal management, the first step is to diagnose the disease, followed by establishing appropriate follow-up and treatment.

This study aimed to determine, through a descriptive survey of physicians, factors that could improve patient care in the DRC. The survey aimed to assess the knowledge and practices of 460 Congolese physicians spread across the country but mainly in three big cities in terms of population, namely, Kinshasa, Lubumbashi, and Mbuji-Mayi, but also smaller cities such as Kananga and Bukavu.

The participation rate was 48%, as observed for similar surveys [16,17]. However, it took over a year to reach this participation rate, requiring diligent follow-up and several reminders. The average age of the physicians was 35 years (range: 25–60 years), as compared to the average healthcare worker’s age of 33 years in 2012 [7]. These results correspond to the 25–54 years age group that constitutes the majority of the population in the DRC [12]. The very low participation of women (18%) might reflect the low level of education of women in the DRC [18]. Physicians who live in the five most populous cities of the DRC, i.e., Mbujimayi (35.4%), Kananga (22%), Kinshasa (13%), Lubumbashi (12%), and Bukavu (12%), are mainly general practitioners (82.4%) [12]. Only 1% (6/460) of them had already received formal training on major sickle cell syndromes. This is not surprising because specialized medical training is limited to a few universities meeting the criteria for postgraduate organizations in the DRC, and these facilities alone cannot absorb all the specialization requests of the country [19]. The physicians also encountered difficulties in organizing the systematic follow-up of sickle cell patients. This is partly due to the local context. In the DRC, for geographical and socioeconomic reasons and due to representations of the disease, patients have little access to health structures. They consult most frequently during complications and most often in health facilities where specialists are not present [20]. However, even these specialists have received little or no specific training on the management of SCD. If the training and health support center (CEFA) based at the Monkole Hospital in Kinshasa organizes training on sickle cell anemia, the sessions are chargeable and do not cover the needs existing throughout the country, while other training course offerings are rare [21]. Health personnel must be regularly trained and equipped for the basic management of SCD, including early detection, crisis prevention, and care, and this concern was raised among the participants [22].

Common diagnostic methods in newborns and beyond are Hb separation techniques like isoelectric focusing, high-performance liquid chromatography, and capillary electrophoresis [23]. In this study, 65% (298/460) of the physicians reported that they had diagnostic difficulties that limited them to clinical diagnosis and biological confirmation only by a sickle cell test. However, the Emmel test is a non-specific test with limits of interpretation, and it is only routinely available in university hospitals and some private laboratories [24]. Newborn screening for SCD was adopted in 1972 in the United States and has spread to other continents [25]. Newborn screening made it possible to identify newborns suffering from SCD before the onset of symptoms, to prevent infectious complications and VOC and to reduce the mortality rate of sickle cell children by up to 84% [25,26]. However, in this study, the circumstances of SCD diagnosis were mainly the presentation of symptomatic patients, and neonatal diagnoses were only reported by 1% of the physicians. This confirmed data in the literature demonstrating that neonatal screening is still very exceptional in sub-Saharan Africa, and so far, no African country has maintained neonatal screening for SCD in its national program [26,27,28]. Currently, rapid tests for screening for sickle cell disease with good sensitivity and good specificity exist [29]. Their use, which could be coupled with rapid malarial tests and a confirmation technique available in reference centers, could also be a strategy to be considered to improve the diagnosis and management of sickle cell patients.

The management of vaso-occlusive crises is not optimal, with more than half of the participants reporting it to be inadequate or insufficient. The same observation was made in Nigeria [22]. Nevertheless, most physicians (95%) believe that the therapeutic means for SCD management are insufficient and unsatisfactory. They point out that there are conditions to be met to improve patient care. In the management of SCD-related pain, it is recommended to provide ample hydration and administer level-three pain relievers such as morphine, in case of uncontrollable pain [30]. However, in the DRC, access to morphine is very limited, and pain management is most often restricted to level-two analgesics. Only physicians who resided in large cities and had less than 10 years of practice had good knowledge of the management of vaso-occlusive crises. Training opportunities in large cities are probably one of the reasons for this better knowledge.

On the other hand, 86% of participants had a good attitude towards acute anemia and recommended blood transfusion. Anemia of any origin is frequent in Africa, which explains why its management is well known. This is essential because acute anemia is responsible for a high mortality rate in sickle cell patients in sub-Saharan Africa [31,32]. Its prevention and effective management dictate specific strategies that are adapted to the African context [24].

The use of hydroxyurea has proven its effectiveness and its long-term tolerance in northern countries [33,34]. Due to its cost, its availability, and the frequently associated risk of male sterility, it is rarely used in Africa. The same observation was made in our study. However, monitoring hydroxyurea treatment and, therefore, the ability to guarantee the biological and clinical controls necessary for monitoring this treatment must not be overlooked. The assurance of regular follow-up, affordability for families, and knowledge of the disease and its treatment by health staff are prerequisites for the initiation of this therapy [24].

Almost all doctors surveyed (99%) were unaware of the different forms of major sickle cell syndromes (SS, SC, Sβ-thalassemia, etc.), and the scarcity of specific training on sickle cell anemia and the lack of organization of this specialization in the DRC can explain this result; on the other hand, the majority of patients in the DRC are homozygous for hemoglobin S [4].

The associations of sickle cell patients were not very well known by the doctors interviewed. Due to a lack of funding, associations carry out little activity and, similar to other pathologies like malaria, their visibility is very reduced because of very little publicity in the DRC.

Physicians’ self-assessments demonstrated that their level of knowledge on SCD was good to medium. However, this self-assessment might not be reliable. For example, 90% of participants reported their absence of knowledge about the major sickle cell syndromes (Sβ-Thalassemia, SC, etc.) and the fact that it was impossible to get a reliable diagnostic test, while most often, the only available test is the Emmel test. In this context, the self-assessment of the respondents on these subjects need to be interpreted with caution. Other studies carried out in sub-Saharan Africa have come to the same conclusions [35]. In Nigeria, the level of care and knowledge of health professionals in the diagnosis and prevention of sickle cell crises is low [22]. Neonatal screening can be a means of improving the knowledge of healthcare teams and helping to prevent complications, as has been shown in an experiment in Burkina Faso and Nigeria [36].

Improvement in sickle cell care can be achieved in different fields or areas. SCD is a serious public health issue in the DRC, but only a few hours are dedicated to the disease in the physician training course content. Para-university training, such as that offered at the national level for HIV and malaria programs, as well as the development of a university diploma program on sickle cell disease, is necessary to strengthen the level of knowledge of healthcare providers. Early accurate diagnosis is important for the prevention of early infection and other causes of death. Inaccurate diagnosis of the disease in individuals with the sickle trait has devastating psychological, economic, social, and medical consequences. However, in resource-limited countries, implementation of a neonatal screening program constitutes a real challenge; the use of new rapid sickle cell tests, which could be coupled with rapid malaria tests, could be another option for all children under 5 years of age with signs of SCD (anemia, infections, pain). The implementation of a reference center that could confirm the diagnosis, for example, by iso-electrofocusing, is also needed. This must be based on past experiences obtained by African teams. One of the other key element for better patient management is patient associations. Supporting and encouraging the creation of associations for sickle cell patients make it possible to ensure various essential actions; among these are the awareness of the population about this disease and support from the government for various concrete actions, such as setting a minimum monthly cost—in the absence of free care—for all sickle cell children to ensure that they are subject to regular follow-up.

## 6. Conclusions

The level of knowledge and practices of Congolese doctors on SCD could be improved. Targeted training on accurate diagnosis and management of SCD were adapted to the local context, both theoretical and practical, which could be combined with existing programs (HIV, malaria), would improve the level of knowledge and optimize sickle cell patient care. The extension of national guidelines for the management of SCD to health workers is needed to help improve the level of knowledge of the disease and the practices for its treatment. The effective involvement of the government is necessary for the creation of diagnostic and referral care centers in each province. Diagnostic tools (rapid sickle cell test) and therapeutic means accessible to patients in fragile socioeconomic situations (folic acid, oral penicillin, anthelmintics, and antimalarials), pain management with morphine, as well as treatment with hydroxyurea must constitute a complementary line of thinking for the training of health personnel.

## Figures and Tables

**Table 1:** Clinical practices and opinions of Congolese physicians about sickle cell disease (*n* = 460).

1. Clinical Practices	*n*	(%)
Do you follow patients who are…?		
(1) Homozygote for HbS (2) Heterozygous for HbS (3) Compound heterozygous for SBeta-thalassemia (4) Compound heterozygous for other sickle cell syndromes	452 4 4 0	(98%) (1%) (1%) (0%)
Do you treat sickle cell patients?		
(1) ≤15 years old (2) >15 years old	452 8	(98%) (2%)
Physicians who prescribe hemoglobin electrophoresis	101	(22%)
Physicians who announce diagnoses to their patients	301	(66%)
Physicians who give appropriate advice to sickle cell patients *	147	(32%)
Physicians who refer patients for psychological support to patient associations	46	(10%)
Physicians who use traditional medicines to treat sickle cell disease	24	(5%)
Physicians who collaborate with NGOs involved in the fight against sickle cell anemia	60	(13%)
Physicians who receive patients referred by traditional healers	24	(5%)
Physicians in possession of the national protocol for the management of sickle cell disease in their structures	67	(15%)
Physicians who know at least a reference center for sickle cell disease in their hometowns	318	(69%)
**2. Opinions of physicians**		
Organization of a university diploma in major sickle cell syndromes in his/her hometown	0	(0%)
Physicians who are aware of their own hemoglobinopathy status	212	(46%)
Physicians who know at least one sickle cell association in their city of residence	78	(17%)
Physicians who think that sickle cell patients are afraid of the disease	386	(84%)
Physicians who think that sickle cell patients keep hope	294	(64%)
Knowledge of sickle cell patients coming from:		
(1) Patient association (2) Support structure (3) Association aid	66 69 124	(14%) (15%) (27%)
Physicians who know the different types of major sickle cell syndromes		
(1) HbSS	454	(99%)
(2) HbSβThal	4	(1%)
(3) HbSC	2	(0%)
(4) HbSOarabic	0	(0%)
(5) HbSDpunjab	0	(0%)
(6) HbSE	0	(0%)

NGOs: non-governmental organizations; HbSS: homozygous S, Composite heterozygote (SC, S-beta thalassemia, SOarabic, SDpunjab, and SE); * rehydration, regular medical follow-up, adherence to prophylaxis (folic acid, oral penicillin, dewormers, and antimalarials), ±1 for advice on factors triggering crisis—intense physical exercises, thermal variations, emotional stress, etc.

**Table 2:** Self-assessment of the level of knowledge of Congolese doctors on hemoglobinopathies.

Parameters	Quotation/5 *
	Sickle Cell Anemia
Advice to patients	3.8
Biological diagnosis	3.7
Clinical diagnosis	3.9
Genetic level	3.2
Pathophysiology map	3.9
Medical management	3.7

* Likert scale.
